# AutoDock VinaXB: implementation of XBSF, new empirical halogen bond scoring function, into AutoDock Vina

**DOI:** 10.1186/s13321-016-0139-1

**Published:** 2016-05-18

**Authors:** Mathew R. Koebel, Grant Schmadeke, Richard G. Posner, Suman Sirimulla

**Affiliations:** Department of Basic Sciences, St. Louis College of Pharmacy, 4588 Parkview Place, Saint Louis, MO 63110 USA; Department of Biological Sciences, Northern Arizona University, S San Francisco St, Flagstaff, AZ 86001 USA

**Keywords:** Halogen bond, Scoring function, Autodock, Vina, Sigma hole, XBSF

## Abstract

**Background:**

Halogen bonding has recently come to play as a target for lead optimization in rational drug design. However, most docking program don’t account for halogen bonding in their scoring functions and are not able to utilize this new approach. In this study a new and improved halogen bonding scoring function (XBSF) is presented along with its implementation in the AutoDock Vina molecular docking software. This new improved program is termed as AutoDock VinaXB, where XB stands for the halogen bonding parameters that were added.

**Results:**

XBSF scoring function is derived based on the X···A distance and C–X···A angle of interacting atoms. The distance term was further corrected to account for the polar flattening effect of halogens. A total of 106 protein-halogenated ligand complexes were tested and compared in terms of binding affinity and docking poses using Vina and VinaXB. VinaXB performed superior to Vina in the majority of instances. VinaXB was closer to native pose both above and below 2 Å deviation categories almost twice as frequently as Vina.

**Conclusions:**

Implementation of XBSF into AutoDock Vina has been shown to improve the accuracy of the docking result with regards to halogenated ligands. AutoDock VinaXB addresses the issues of halogen bonds that were previously being scored unfavorably due to repulsion factors, thus effectively lowering the output RMSD values.

**Electronic supplementary material:**

The online version of this article (doi:10.1186/s13321-016-0139-1) contains supplementary material, which is available to authorized users.

## Background

Molecular docking is a widely used computational chemistry technique in the structure-based drug design process [1, 2]. Molecular docking is used to predict and rank the bound conformation of protein–ligand complexes and their binding affinities. Two main rudimentary components of docking programs include a scoring function and a search algorithm. The binding affinities (ΔG) are theoretically calculated using the programs predefined scoring function based on the given interactions associated with each conformation. The scoring functions in most programs are used to evaluate the contacts between protein and ligand atoms for each binding pose and rank them based on their noncovalent interactions such as hydrogen bonds, nonpolar–nonpolar contacts (van der Waals), repulsion forces and solvation parameters. Another non-covalent interaction is halogen bonding, which is now widely recognized as an important interaction in the protein–ligand complexes. Most pharmaceutical drugs are halogenated and are capable of forming halogen bonds with the biomolecules. Halogens in organic molecules are classically perceived to possess electronegative charges, however they are known to possess both electronegative and electropositive charges on them. The electropositive potential on the halogen atom is usually referred to as the σ-hole [3], which leads to the formation of a halogen bond with an electronegative atom. A halogen bond in biological molecule can be referred to as a short C–X···A–Z interaction, where A is a halogen bond acceptor, C–X is a carbon-bonded halogen (chlorine, bromine, or iodine), and A–Z is an electron pair donor group such as carbonyl, hydroxyl, thiol, aromatic ring, charged carboxylate, phosphate group, or amine [4, 5]. In molecular mechanical approaches, atoms are usually defined as an atom type and a partial charge. Since halogens have anisotropic electron charge distribution around it, the usual force fields used in molecular mechanics fail to account for halogen bonding contributions. Recently, some approaches were developed to address the σ-hole effect of halogens (Cl, Br, and I) in molecular mechanical calculations [6–13]. Ibrahim et al. first introduced it in AMBER, and then Jorgensen et al. added extra sites (X-sites) to OPLSA-AA force field to address halogen bonding and implemented it in BOMB, MCPRO and BOSS programs [6, 9]. Prof. P Shing Ho’s group has recently developed *ffBXB* force field for treatment of halogen bonding in AMBER [11]. Currently, most docking programs do not account for the presence of halogen bonding in their scoring functions. Hence, integration of halogen bonding potentials in the docking scoring function would be highly beneficial in achieving accuracy of docking results with respect to halogenated ligands. Hobza et al. introduced halogen bonding parameters into a docking scoring function by adding massless positive point charge (dummy atom) to the halogen atoms to represent σ-holes in the UCSF DOCK program [14]. Zhu et al. derived a knowledge based scoring function called XBPMF, which is independent of dummy atoms [15]. However, currently available crystallographic data on halogen bonds in the PDB is inadequate to accurately define a knowledge-based scoring function. Very recently Böckler et al. published a QM derived empirical scoring function for the interaction between aromatic halogenated ligands and the protein backbone carbonyl oxygen atom [16]. Here, we present a more accurate empirical scoring function for halogen bonding, which is termed as “XBSF” along with its implementation in AutoDock Vina. The newly defined scoring function is not limited to just the backbone carbonyl oxygen. It considers oxygen, nitrogen and sulfur as the halogen bond acceptors. It can also be easily extended to π systems. However it should be noted that backbone carbonyl oxygen is usually the major contributor to halogen bonding in protein–ligand systems. Additionally, it would be more practical for virtual screening applications. AutoDock Vina was chosen for implementation of our scoring function as it is one of the most widely used free docking software program. Henceforth, the new docking software is designated as VinaXB.

## Design and implementation

### Halogen bond scoring function (XBSF)

In this paper, an empirical scoring function for halogen bonding is presented along with its implementation in AutoDock Vina. An approach similar to that of hydrogen bonding in X-CSCORE was used in the development of this scoring function [17]. However, more parameters were added to address the shape, size and anisotropic charge of the halogen atoms. In Vina, the hydrogen bonding term is based on d, where d is the overlap of van der Waals radii of interacting atoms. The value for d is calculated by subtracting the sum of the van der Waals radii of interacting atoms from the internuclear distance of interacting atoms as described in Jain [18]. Hydrogen bonding term equals 1 when d < −0.7 Å; 0, when d > 0 Å, and linearly interpolated in between these distances. In XBSF, to define the halogen bonding term, a similar approach was incorporated, however, due to the anisotropic charge on halogen, an angle term was included to account for the varying positive charge on the atom. The XBSF scoring function (**E**) is defined using these three terms: weight, angle factor, and distance factor as follows in Eq. (1):

1 $${\text{E}} = {\text{W}}\Phi {\text{D}}$$

where W = weight, Φ = angle factor, D = distance factor

### Weights (W)

The halogen weights used in the program were adapted from the energy well (εx) calculations by Scholfield et al. (Cl is −0.265, Br is −0.32, and I is −0.4) [19]. All halogen bond acceptors (O, N, and S) are equally weighted so that no additional parameters are necessary.

### Angle factor (Φ)

**Fig. 1:**
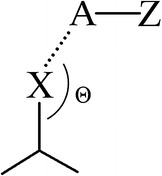
Representation of C–X···A angle (Θ) that pertains to halogen bonding, where X = Cl, Br, I and A = O, N, S

The sigma hole on the halogen is more prominent on the distal end of the C–X bond and the positive charge decreases as the C–X···A angle (as shown in Fig. 1 as Θ) deviates from 180. So when calculating the angle factor, which is dependent on the effective charge at the point of interaction with the halogen, the equation developed by Scholfield et al. was used [19]. The equations to calculate the effective charge and angle factor at each angle on the halogen are given below:

2 $$Z_{X,\varTheta } = A\cos \left( {\nu \alpha } \right) + B$$

$$where\; \alpha = 180^\circ - \varTheta$$

3 $$\Phi = \left\{ {\begin{array}{*{20}l} {\frac{{Z_{{{\text{X}},\Theta }} }}{{Z_{X,180} }}\quad for\;\Theta _{0} \le\Theta \le 180^\circ } \hfill \\ { 0\quad otherwise } \hfill \\ \end{array} } \right.$$

**Fig. 2:**
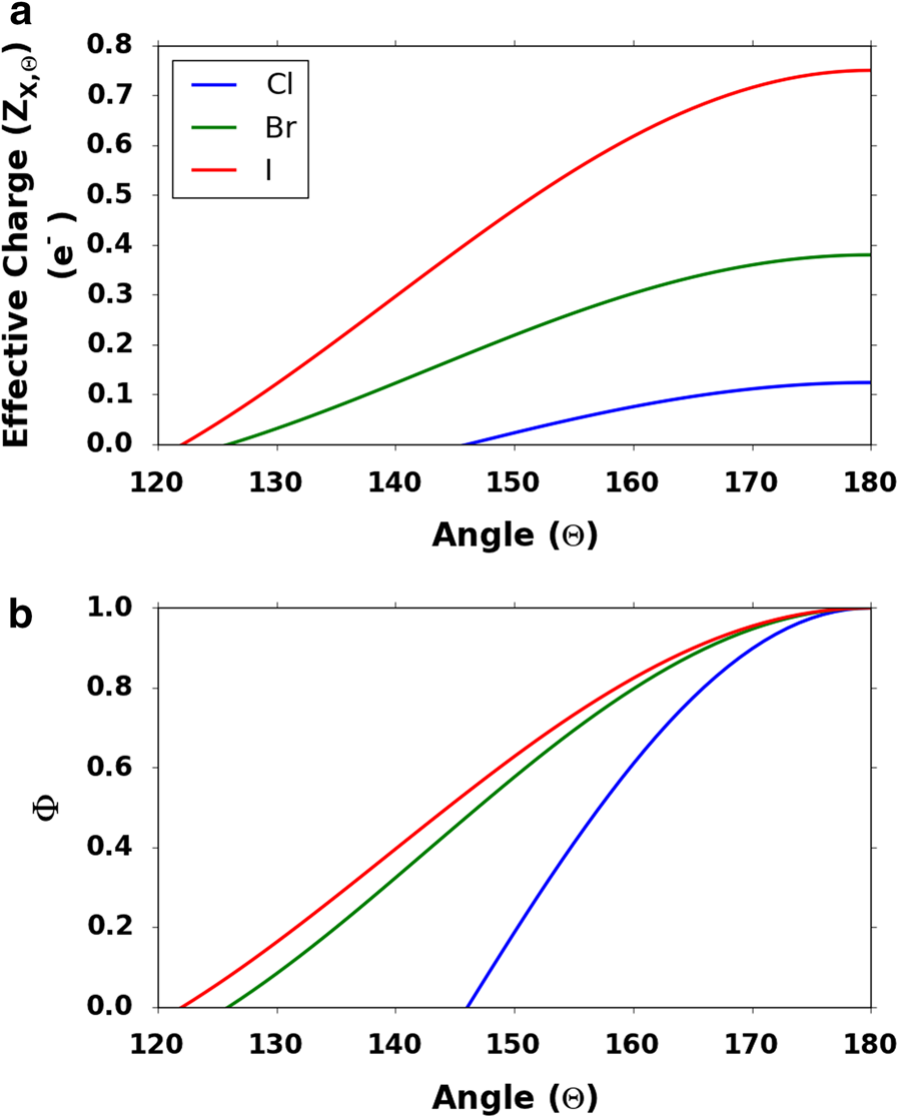
**a** Effective charge (Z_X,Θ_) to angle (Θ) ratio. **b** Angle factor (Φ) to angle (Θ) ratio

*Z*_X,Θ_ is the effective charge on the halogen at Θ angle. The effective charge on halogens at various angles is shown in the Fig. 2a. It is important to note that the electroneutral points (Θ_0_) which is where the charge transitions from partial positive to partial negative for the halogens are different depending on the type of halogen as shown in Table 1 [19].

**Table 1:** The Electroneutral angle (Θ_0_) values of halogens

Halogen	Electroneutral angle (Θ_0_)
CL	146°
Br	126°
I	122°

### Distance factor (D)

**Fig. 3:**
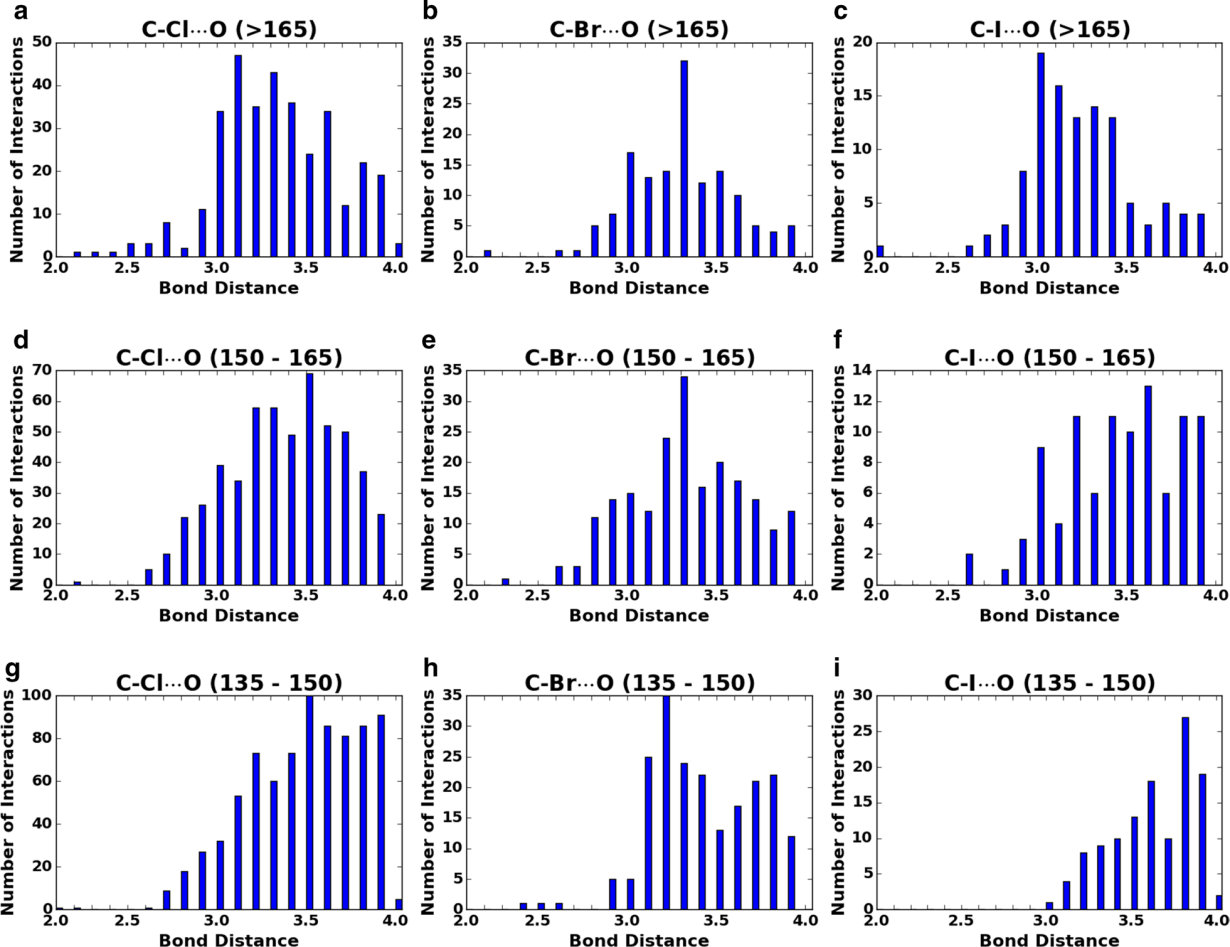
Number of halogen bond interactions observed in bond angle regions. **a**, **d**, **g** Represent the number of interactions of C–Cl···O at angles 165°–180°, 150°–165°, 135°–150° respectively. **b**, **e**, **h** Represent the number of interactions of C–Br···O at angles 165°–180°, 150°–165°, 135°–150° respectively. **c**, **f**, **i** Represent the number of interactions of C–l···O at angles 165°–180°, 150°–165°, 135°–150° respectively

In order to accurately incorporate the distance factor, we had to carefully study the size and shape of the halogens. The shapes of the halogens are known to be aspherical which is caused by the polar flattening at their σ-hole end [19]. Due to this effect, the radius of the atom is decreased at the σ-hole end and radius would bulge in the position orthogonal to the sigma hole. A study of quantum mechanical MP2 level calculations of distance–angle relationship of He with Br_2_ clearly supports the polar flattening effect [10]. In this study, we examined the polar flattening effect in the crystal structures of the protein–ligand complexes. Here, we mined the latest PDB release from www.wwpdb.org (Feb 2015) for C–X···O interactions using the sigmahole.py script which was published previously [5]. The collected data is graphically displayed in Fig. 3, and was used to determine the optimal radii overlap (δ value).

We separated the data of C–X···O interactions by 15° increments from 135° to 180° to spot patterns in the overlap distance at different angles and to identify the optimal distances in a manner similar to how Wang et al. established their cutoff distances for hydrogen bonding in X-CSCORE [17]. The data from the Fig. 3 shows a clear trend for I···O and Cl···O. The distances for I···O interactions peaked between 3.1 and 3.4 Å for 165°–180°, and they shifted to 3.7–3.9 Å for 135°–150°. A Similar trend is also observed for Cl···O interactions where distances are peaked ~ 3.1 Å for 165°–180° and shifted to ~ 3.5 Å for 135°–150°. For Br···O interactions, a shift in the optimal distance was not evident, however there is an evidence of increased interactions in the range of 3.6–3.8 Å for 135°–150° compared to 165°–180°. Overall, the data suggests there is decrease in radii near the σ-hole angle. Based on the analysis of the data above, the distance term (D) is defined as follows:

The distance term (D) equals 1, when D < −δ Å, equals 0, when D > 0, and is linearly interpolated in between as shown in Table 2.

**Table 2:** The δ value of halogens per given angle ranges

Halogen	Angle range	δ value (Å)
Cl	165°–180°	0.25
Cl	150°–164°	0.15
Br	165°–180°	0.45
Br	150°–164°	0.35
Br	135°–149°	0.25
I	165°–180°	0.55
I	150°–164°	0.45
I	135°–149°	0.35

The radii values, Table 3, used to calculate the overlap are the same as the ones already present in the Vina program to maintain consistency.

**Table 3:** The van der Waals radii (Å) values of associated atoms

Atom	Van der Waals radii (Å)
Cl	1.8
Br	2.02
I	2.2
O	1.7
N	1.8

### PDB analysis

The latest PDB release (Feb. 2015) was obtained from www.wwpdb.org and the files containing halogenated ligands were extracted similar to our previous study [5]. The separated files had a resolution of 3 Å or less and at least one C–X bond. C–X···A interactions were calculated using sigmahole.py script [5].

### Docking

The ligands were removed from the PDB crystal structures, and then the protein was prepared using the prepare_receptor4.py script from AutoDock tools [20]. This process starts by removing the waters present, then adds polar hydrogens, and finishes by removing any nonstandard residue ligands. Ligands were downloaded separately from the rcsb.org website and prepared using mgltools [20]. A custom made script was written to calculate the grid box of the ligand PDB files by taking the maximum and minimum values of the x, y, and z coordinates and adding a total of 15 Å per each axis. In addition to expanding the grid box we also instituted a randomized shift of the grid box in any, all, or none of the directions by a measurement of 2.5 Å. The purpose of the grid box manipulations was to ensure a fair and accurate testing of the two programs regardless of centering of the grid box. A similar manipulation was done for the testing of AutoDock Vina [21]. A seed is a randomly generated number used by the AutoDock Vina program for its starting position and parameter of the search [21]. The seed used for each of the dockings in this study were generated by the python random number generator and assigned to config.txt files. The config.txt files contain the receptor file name, the ligand file name, the seed, the x, y and z coordinate of the center of the grid box and the size of the grid box. We used the same config.txt files for each complex in both Vina and VinaXB dockings to ensure the same parameters for comparison. The proteins and ligands were then docked and scored in both Vina and VinaXB. The script used to calculate RMSD values are attached as the Additional file 1.

## Results and discussion

**Fig. 4:**
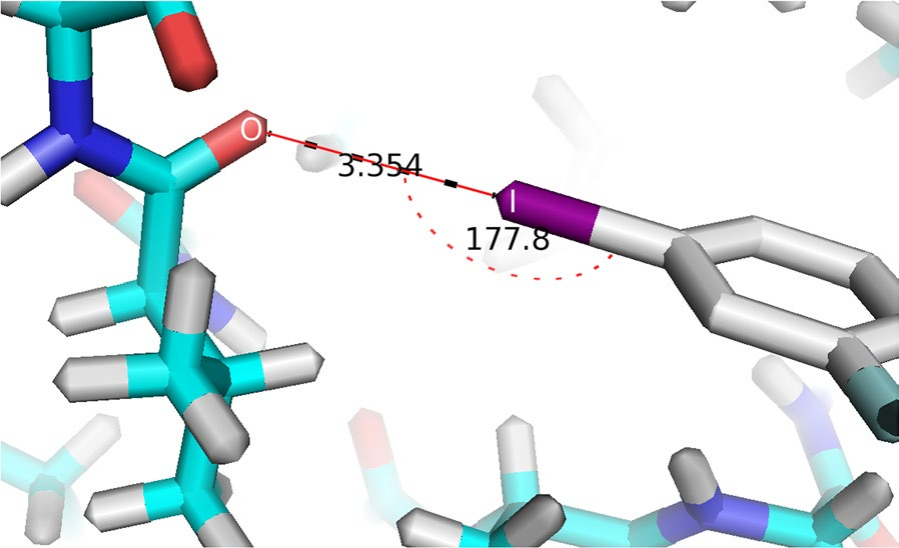
Halogen bond formation in PDB 3DY7 between the backbone carbonyl oxygen of the protein and the iodine of the ligands

The above described scoring function was successfully utilized to test 106 halogenated ligand–protein complexes from the PDB that are known to form halogen bonds. One such example is the PDB 3DY7 (structure of human mitogen protein kinase 1 (MEK1) with the (5S)-4,5-difluoro-6-[(2-fluoro-4-iodophenyl)imino]-N-(2-hydroxyethoxy)cyclohexa-1,3-diene-1-carboxamide compound as an inhibitor), which possesses a halogen bond interaction formed between the iodine of the ligand and the backbone carbonyl oxygen of the protein as illustrated in Fig. 4 by the red line [22]. XBSF scoring function calculates the halogen bonding term C–I···O interaction as follows. The C–I···O interaction angle is measured as 177.8°, by using the Eqs. (2) and (3) the angle term (Φ) is derived as a value of 0.998. The distance of the I···O bond is 3.354 Å which gives a van der Waals radii overlap (δ) of 0.546 Å. This value for the overlap is used to calculate the distance term (D) of 0.992, which is referenced to Table 2.

**Fig. 5:**
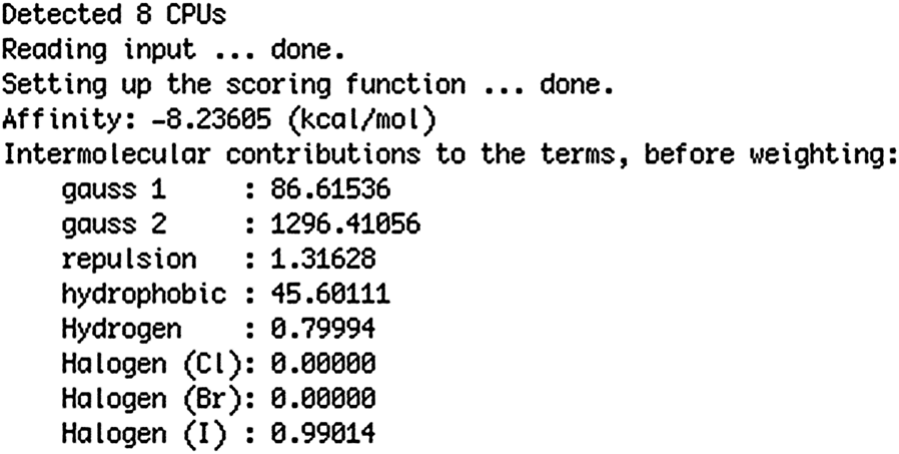
Score only output for PDB 3DY7 from VinaXB prior to weighing

Using Eq. (1) and the given value for the distance term (0.992) and angle term (0.998) previously mentioned, both of which have been rounded to three significant digits, XBSF calculated the pre-weight score only of 0.99014 out of a potential 1.00000 as displayed below in Fig. 5.

**Fig. 6:**
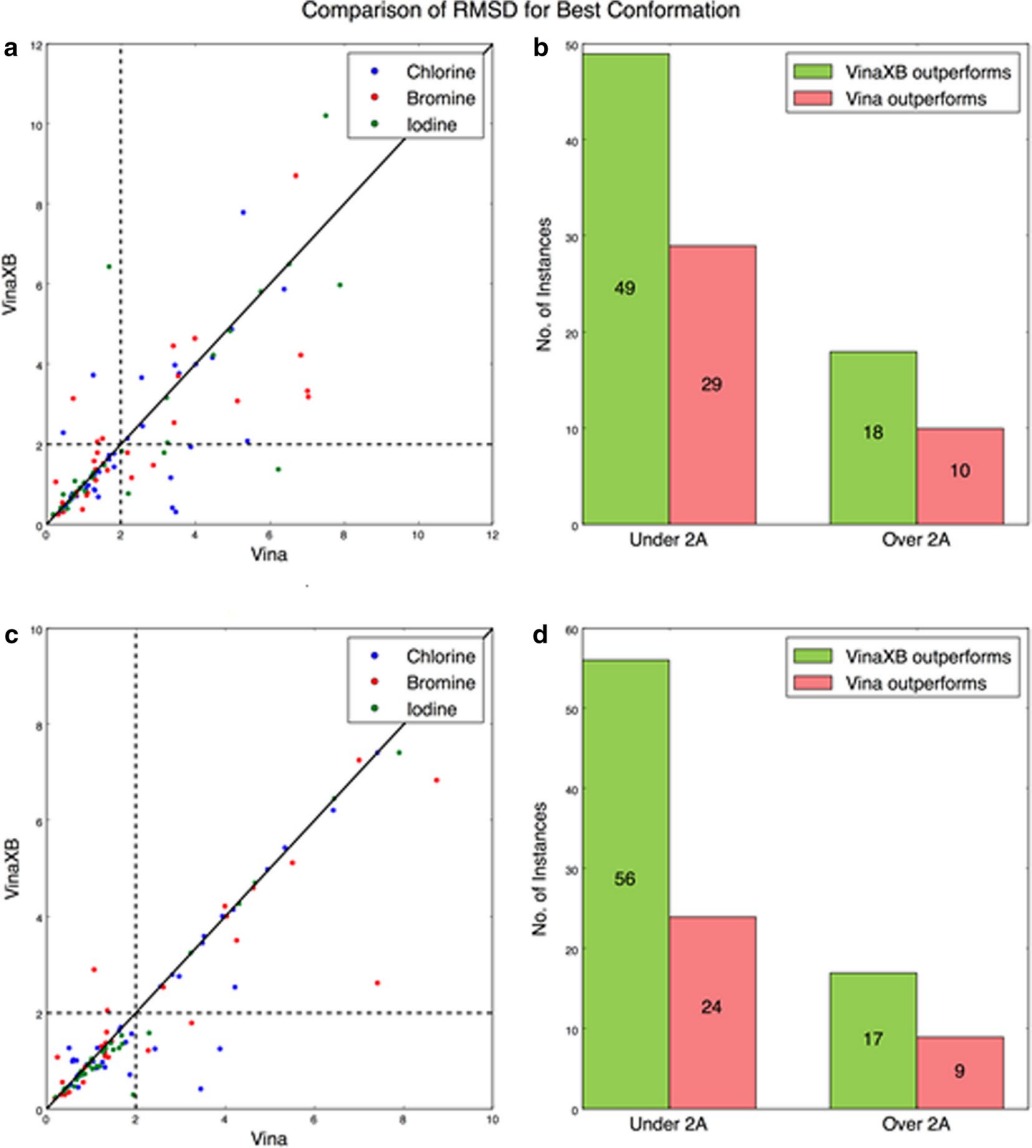
**a** Scatter plot comparison of RMSD values of the best conformations using Vina versus VinaXB at exhaustiveness 8. **b** Bar chart showing the number of instances in which Vina or VinaXB performed better than the other, when RMSD values are above and below 2 Å at exhaustiveness 8. **c** Scatter plot comparison of RMSD values of the best conformations using Vina versus VinaXB at exhaustiveness 100. **d** *Bar chart* showing the number of instances in which Vina or VinaXB performed better than the other, when RMSD values are above and below 2 Å at exhaustiveness 100

The newly derived halogen bonding scoring function is compared with the Vina scoring function using a set of 106 protein-halogenated ligand complexes. Of these, there are 39 chlorinated ligands, 32 brominated ligands, and 35 iodinated ligands present. The PDB codes of the files used in the study are attached in the Additional files 2, 3, and 4. We performed the docking calculations at default exhaustiveness (exhaustiveness of eight) which in Vina controls the number of evaluations during each local optimization, starting from random conformations [21]. Figure 6 illustrates the RMSD (root-mean-square deviation) values of the best conformations in respect to the lowest distance from the native conformation of the ligand for each dock. The total 106 halogen bonding protein ligand complexes that were ran through both Vina and VinaXB were compared based on their RMSD values with respect to the native ligand conformation. For each complex, the program (Vina or VinaXB) that resulted with the lower RMSD value was considered to be the winning one. The docking was performed at the standard exhaustiveness to compare the accuracy of the programs where most users will be docking. During the trials, VinaXB performed superior to Vina. For the given set, at exhaustiveness eight, VinaXB had 49 conformations with RMSD less than 2 Å whereas Vina only managed to get 29 with the RMSD values under 2 Å. It should be noted that the quantity of RMSD values below 2 Å got better in concurrence with the increase in exhaustiveness. The performance of VinaXB found to be much superior when the docking was performed at higher exhaustiveness such as exhaustiveness 100, where the p value was calculated to be 0.0037. The RMSD values and the statistical results are available in Additional files 5, 6, 7, and 8. Figure 6b, d shows the instances in which either Vina or VinaXB performed better than the other. From the results it is evident that VinaXB performed better than Vina (for the given set of compounds).

An attempt was made to compare the docking results of VinaXB with the experimental values for halogenated ligand pdb files. However, there were only 26 halogenated pdb files in the PDBBind database [23, 24] that contain experimental (kd/ki) values. Since the data was not large enough to make a statistically significant comparison, this data was not used the for our analysis. We have attached the data of pdb ID’s and their corresponding experimental values as an Additional file 9. 

## Conclusion

XBSF, a new and accurate empirical scoring function for scoring halogen bonds has been defined. Its incorporation into the Vina program has been proven successful by the ability to locate a high percentage of top conformations with a lower RMSD than the original Vina. Along with VinaXB, XBSF is also suitable for a wide array of molecular docking programs and the virtual screening process. The increased accuracy of VinaXB came with no change to the user interface in comparison to Vina. VinaXB has no effect on non-halogenated ligand complexes, as there are no alternations to any parameters of Vina outside the identification of halogen bonds. With the ever growing usage of halogens in the drug design optimization stage, VinaXB will be an indispensable tool to medicinal chemists.

## Software availability and requirements

Project name: AutoDock VinaXB. Project home page: http://www.sirimullaresearchgroup.com/software.html. Operating system(s): Mac, Linux, Windows. Programming language: C++. Other requirements: Boost Library. License: Apache 2.0. Any restrictions to use by non-academics: no license needed. VinaXB is now available to download for free at https://github.com/ssirimulla/vinaXB as well as at www.sirimullaresearhgroup.edu.

## Additional files

10.1186/s13321-016-0139-1 RMSD program.
10.1186/s13321-016-0139-1 PDB IDs that has halogen bond using Bromine.
10.1186/s13321-016-0139-1 PDB IDs that has halogen bond using Chlorine.
10.1186/s13321-016-0139-1 PDB IDs that has halogen bond using Iodine.
10.1186/s13321-016-0139-1 RMSD values at exhaustiveness 8.
10.1186/s13321-016-0139-1 RMSD values at exhaustiveness 100.
10.1186/s13321-016-0139-1 Statistical results at Exhaustiveness 8.
10.1186/s13321-016-0139-1 Statistical results at Exhaustiveness 100.
10.1186/s13321-016-0139-1 Halogen pdbbind energy.

## Abbreviations

d: sum of van der Waals radii in Å of the interacting atoms
XBSF: halogen bond scoring function
MP2: second-order Møller–Plesset
QM: quantum mechanics
