# Supplementary material for: AutoDock VinaXB: implementation of XBSF, new empirical halogen bond scoring function, into AutoDock Vina
Source: J Cheminform. 2016 May 18;8:27. doi: 10.1186/s13321-016-0139-1 (PMC4870740; doi:10.1186/s13321-016-0139-1)
Supplement: Supplementary file 7 — 10.1186/s13321-016-0139-1 Statistical results at Exhaustiveness 8. [file 13321_2016_139_MOESM7_ESM.pdf]

Signed Rank Test for Vinaxb and Vina, calculated off the difference score Vinaxb-Vina=Diff

The UNIVARIATE Procedure
Variable: diff

Moments			
N	106	Sum Weights	106
Mean	-0.1934272	Sum Observations	-20.503288
Std Deviation	1.28548048	Variance	1.65246007
Skewness	-0.3968439	Kurtosis	4.25844178
Uncorrected SS	177.474202	Corrected SS	173.508307
Coeff Variation	-664.58087	Std Error Mean	0.1248569

Basic Statistical Measures			
Location		Variability	
Mean	-0.19343	Std Deviation	1.28548
Median	-0.03078	Variance	1.65246
Mode	.	Range	9.60960
		Interquartile Range	0.33905

Tests for Location: Mu0=0				
Test	Statistic		p Value	
Student's t	t	-1.54919	Pr > |t|	0.1243
Sign	M	-14	Pr >= |M|	0.0084
Signed Rank	S	-751.5	Pr >= |S|	0.0171

Quantiles (Definition 5)	
Level	Quantile
100% Max	4.7619127
99%	2.7043606
95%	2.0235827
90%	0.6960297

Signed Rank Test for Vinaxb and Vina, calculated off the difference score Vinaxb-Vina=Diff

The UNIVARIATE Procedure
Variable: diff

Quantiles (Definition 5)	
**Level**	**Quantile**
**75% Q3**	0.0444254
**50% Median**	-0.0307785
**25% Q1**	-0.2946197
**10%**	-1.8982790
**5%**	-2.9657658
**1%**	-3.8472232
**0% Min**	-4.8476908

Extreme Observations			
**Lowest**		**Highest**	
**Value**	**Obs**	**Value**	**Obs**
-4.84769	106	2.42230	62
-3.84722	64	2.45893	33
-3.69770	49	2.49136	26
-3.31856	38	2.70436	99
-3.16145	6	4.76191	104
