# Supplementary material for: AutoDock VinaXB: implementation of XBSF, new empirical halogen bond scoring function, into AutoDock Vina
Source: J Cheminform. 2016 May 18;8:27. doi: 10.1186/s13321-016-0139-1 (PMC4870740; doi:10.1186/s13321-016-0139-1)
Supplement: Supplementary file 8 — 10.1186/s13321-016-0139-1 Statistical results at Exhaustiveness 100. [file 13321_2016_139_MOESM8_ESM.pdf]

Signed Rank Test for Vinaxb and Vina, calculated off the difference score Vinaxb-Vina=Diff on RMSD Data

The UNIVARIATE Procedure
Variable: diff

Moments			
N	106	Sum Weights	106
Mean	-0.2154804	Sum Observations	-22.840921
Std Deviation	0.74617523	Variance	0.55677747
Skewness	-3.1579204	Kurtosis	15.7890138
Uncorrected SS	63.3834047	Corrected SS	58.4616343
Coeff Variation	-346.28453	Std Error Mean	0.07247494

Basic Statistical Measures			
Location		Variability	
Mean	-0.21548	Std Deviation	0.74618
Median	-0.03315	Variance	0.55678
Mode	.	Range	6.62380
		Interquartile Range	0.25432

Tests for Location: Mu0=0				
Test	Statistic		p Value	
Student's t	t	-2.97317	Pr > |t|	0.0037
Sign	M	-20	Pr >= |M|	0.0001
Signed Rank	S	-1278.5	Pr >= |S|	<.0001

Quantiles (Definition 5)	
Level	Quantile
100% Max	1.8301727
99%	0.8379079
95%	0.4092166
90%	0.2075898

Signed Rank Test for Vinaxb and Vina, calculated off the difference score Vinaxb-Vina=Diff on RMSD Data

The UNIVARIATE Procedure
Variable: diff

Quantiles (Definition 5)	
**Level**	**Quantile**
**75% Q3**	0.0128162
**50% Median**	-0.0331516
**25% Q1**	-0.2415053
**10%**	-0.7655052
**5%**	-1.6469397
**1%**	-3.0428478
**0% Min**	-4.7936242

Extreme Observations			
**Lowest**		**Highest**	
**Value**	**Obs**	**Value**	**Obs**
-4.79362	49	0.438189	21
-3.04285	1	0.686546	42
-2.63492	33	0.774384	15
-1.90671	52	0.837908	71
-1.68234	3	1.830173	44
